# Effectiveness of a multidisciplinary team-delivered deprescribing intervention for patients with chronic kidney disease: a protocol for a randomised controlled trial

**DOI:** 10.1080/20523211.2025.2588928

**Published:** 2025-11-26

**Authors:** Amani Zidan, Abdullah Hamad, Rania Ibrahim, Mohamed El-Kadi, Hasan El-Malki, Mohamed Al-Esnawi, Safeya Habib, Muhammad Abdul Hadi, Fatema Babiker, Daoud Al-Badriyeh, Ahmed Awaisu

**Affiliations:** a QU Health, Qatar University, Doha, Qatar; b Nephrology Division, Hamad Medical Corporation, Doha, Qatar; c College of Pharmacy, QU-Health Sector, Qatar University, Doha, Qatar

**Keywords:** Deprescriptions, chronic kidney disease, renal dialysis, clinical trial, kidney failure

## Abstract

**Introduction:**

Inappropriate polypharmacy is prevalent among patients with chronic kidney disease (CKD), and can be mitigated by deprescribing. We aim to evaluate the clinical and economic impact of a multidisciplinary deprescribing programme in Qatar’s healthcare system.

**Methods:**

This randomised controlled trial will be conducted at ambulatory dialysis centres, with an internal pilot to assess the feasibility of recruitment, intervention implementation, and safety measures. Patients will be randomised to usual care or an intervention group where a clinical pharmacist, in collaboration with a multidisciplinary healthcare team, will deliver a structured deprescribing intervention, including periodic monitoring over six months. Outcomes will be measured at baseline, three, and six months. The primary outcome is the proportion of patients with at least one potentially inappropriate medication (PIM). Secondary outcomes include pill burden, treatment burden, hospitalisations, emergency department visits, quality of life, and adherence. A cost–benefit analysis will also be performed. A total of 250 patients provides 90% power to detect a 50% reduction in PIM prevalence (α = 0.05). The statistical analysis will be based on the intention-to-treat principle, using mixed-effects models to account for repeated measures.

**Discussion:**

Findings will provide novel evidence on deprescribing in CKD, informing future clinical and policy interventions.

**Trial Registration:**

ClinicalTrial.gov. NCT06324045. Registered on 15 March 2024.

## Introduction

Chronic kidney disease (CKD) is a major global public health problem, with an estimated 11.7–15.1% of the global population affected.(Lv & Zhang, 2019) Patients with CKD often experience multiple complications and comorbidities, including cardiovascular diseases (CVDs), anemia, diabetes, and thyroid disorders (Fraser et al., 2015). The management of CKD typically leads to polypharmacy, as most disease management approaches and clinical practice guidelines often address these comorbidities and complications in isolation, rather than considering their interrelatedness (Fraser et al., 2015; George et al., 2021; Manski-Nankervis et al., 2021).

Polypharmacy can often be inappropriate, and it is associated with several adverse consequences on patient health outcomes (Johansson et al., 2016). These consequences include, but are not limited to, deterioration in quality of life, increased hospitalisations, falls, and higher mortality rates (Ernst et al., 2020). Inappropriate polypharmacy is particularly prevalent among patients with CKD (Triantafylidis et al., 2018). For example, a study found that 45% of old veterans in the US with kidney disease were prescribed one or more inappropriate or incorrectly dosed medications (Chang et al., 2015). Similarly, in Qatar, where approximately 13% of the population suffers from CKD (Reddy & Cho, 2016), a pharmacoepidemiologic study reported that 75% of older adults were prescribed five or more medications (Al-Dahshan et al., 2020). Moreover, medication-related burden has been linked to decreased medication adherence among older adults in Qatar (Zidan et al., 2018).

A prospective, cross-sectional study was conducted to assess treatment-related burden and health-related quality of life (HR-QOL) among 280 patients diagnosed with CKD at a tertiary care kidney centre in Qatar (Al-Mansouri et al., 2021). Nearly one-third of the participants, especially those receiving hemodialysis, reported a moderate to high treatment-related burden. HR-QOL scores were significantly lower in patients receiving hemodialysis (HD) compared to those who were pre-dialysis. The study highlighted the negative impact of higher treatment-related burden on the patient’s quality of life. The investigators recommend that healthcare systems and clinicians account for this burden when developing CKD management strategies. Some of these strategies could include medication reconciliation and deprescribing (Al-Mansouri et al., 2021).

Deprescribing is a proactive intervention designed to minimise the adverse consequences of inappropriate polypharmacy (Reeve et al., 2017). It involves the supervised process of stopping a medication, reducing its dose, or replacing it with a safer alternative in order to improve patients outcomes and reduce adverse drug events (ADEs) (Gazarin et al., 2020). Several studies have demonstrated the safety and potential effectiveness of deprescribing in improving quality of life and medication adherence, as well as reducing ADEs, mortality rates, and potentially inappropriate medications (PIMs) (Gazarin et al., 2020; Page et al., 2016; Reeve et al., 2017; Romano et al., 2022; Thio et al., 2018). Inappropriate polypharmacy also places a burden on healthcare systems, incurring additional costs from managing the associated ADEs. Implementing deprescribing programmes can help reduce these costs, including those related to admissions due to ADEs and unnecessary drug therapy (Hurley et al., 2024; Kemp et al., 2011; Kutner et al., 2015; Scott et al., 2015).

Although deprescribing protocols and guidelines are now widely available (Manski-Nankervis et al., 2021; Reeve, 2020), their implementation in clinical practice remains limited (Farrell et al., 2015; Scott et al., 2017). Deprescribing is rarely implemented in clinical practice due to several barriers (Okeowo et al., 2023), including a lack of awareness among healthcare providers (Doherty et al., 2020), limited time during consultations (Doherty et al., 2020), insufficient training on deprescribing protocols (Doherty et al., 2020), and resistance from patients who may be hesitant to discontinue long-term medications (Okeowo et al., 2023). Furthermore, there is still a need for robust studies to provide strong evidence of the impact of these programmes on various health outcomes (Rankin et al., 2018). Given the high prevalence of polypharmacy among patients with CKD, there is an urgent need to implement and evaluate promising strategies such as deprescribing for this population (Manski-Nankervis et al., 2021; Triantafylidis et al., 2018). We believe deprescribing is a prudent and innovative intervention that could yield significant clinical and economic benefits if effectively integrated into the Qatar healthcare system. We hypothesise that patients with stage 5 CKD, especially those receiving HD or managed in low-clearance clinics, represent ideal candidates for deprescribing interventions.

The aim of the current study is to conduct an RCT, including an internal pilot, to compare the clinical outcomes and cost–benefit of a multidisciplinary team-delivered deprescribing intervention with usual care. This study represents the evaluation stage of a larger programme, divided into multiple stages, following the Medical Research Council (MRC) guidance on the development and evaluation of complex interventions (Skivington et al., 2024).

## Methods and analysis

### Study design

**Figure 1.**
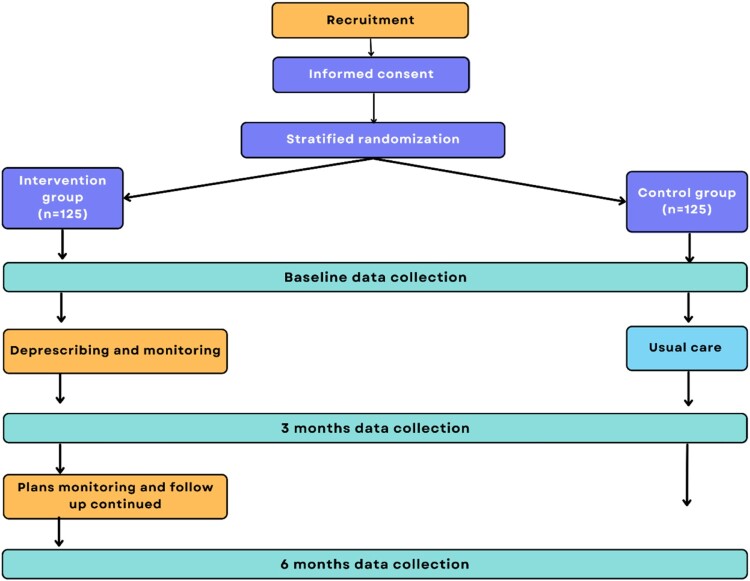
Flowchart of the proposed randomised controlled trial.

This is a pragmatic, multicenter, randomised controlled trial (RCT) with a parallel-group design and an embedded internal pilot. It is being conducted in ambulatory dialysis centres at Hamad Medical Corporation (HMC) to evaluate the effectiveness of a multidisciplinary team-delivered deprescribing intervention on clinical, humanistic, and economic outcomes. The internal pilot aims to assess the feasibility of recruiting a sufficient number of CKD patients, providing the intervention components effectively, and ensuring that there are no safety concerns associated with the intervention. The study is currently recruiting participants and is expected to be completed by 2026. Figure 1 illustrates the overall study design.

### Study participants

The study population includes patients with CKD who have regular follow-up appointments at any HMC-affiliated kidney centre. Patients with CKD undergoing HD three times per week and pre-dialysis patients with low renal clearance who attend monthly follow-up appointments will be recruited for the study. Individuals with eGFR < 15 mL/min/1.73 m^2^, who have not yet reached the threshold for HD are usually managed in these clinics. Patients who require HD but have declined the procedure will not be included in the study.

#### Inclusion criteria

- Patients diagnosed with kidney failure (KF), receiving HD treatment or pre-dialysis patients (eGFR <15 mL/min/1.73 m²) attending a low clearance clinic.
- Patients receiving care at one of the ambulatory kidney centres in Qatar for at least two months.
- Patients able to communicate in Arabic and/or English.

#### Exclusion criteria

- Life expectancy of less than 6 months.
- Unstable or have a diagnosed psychiatric condition.
- Exhibiting uncontrolled or exit-seeking behaviours (e.g. attempting to leave the premises due to confusion, frustration, or anger).
- Critically-ill patients, pregnant women, children, individuals with mental illness, dementia, and unconscious patients.

### Study setting

The study setting is the ambulatory dialysis centres in Qatar, which are operated by HMC. The centres include Fahad Bin Jassim, Al-Wakrah, Shahania, and Alshamal Kidney Centres, which provide dialysis and pre-dialysis care for Qatari nationals and other residents of Qatar with CKD. In addition to dialysis services, the centres also offer outpatient pre-dialysis care for patients with advanced CKD. In addition, different specialty teams, including cardiology, endocrinology, vascular surgery, and psychology, provide consultations and care for the patients at these facilities.

### Recruitment

CKD patients are screened for eligibility through medical records. Eligible patients are invited to participate during their routine visits to an ambulatory kidney centre in Qatar. To ensure feasibility and practicality, selecting patients follows a non-probabilistic approach, recruiting consecutive cases as they become available. Recruitment interaction happens at the beginning or end of the appointment times to minimise disruptions and avoid additional burden to patients while receiving treatments. The recruitment process is performed by trained research assistants.

### Study groups

The study comprises two groups: (1) Control arm: Patients who receive usual (i.e. standard) ambulatory care. This entails monthly medication reviews conducted as part of routine care, mainly by the nephrologist and in-charge nurses. Moreover, any issues highlighted by the patients or the care team are addressed by the attending physician or referred for necessary consultations. The pharmacist's role in these rotations is limited and occasional, with limited MDT discussions including the patient's medications. Importantly, the setting is no different than other HD and clinic settings where usually the changes of the medications are subject to present complaints or apparent health problems; (2) Intervention arm: Patients who receive a structured deprescribing intervention delivered by the multidisciplinary team during their patient daycare visits at the centre.

### Multidisciplinary deprescribing team for chronic kidney disease

The multidisciplinary deprescribing team for CKD (MDT-CKD) includes one nephrologist, two consultants, two clinical pharmacists, two nurses, and one clinical nurse specialists. This team is restricted to the intervention arm to prevent contamination. The team members are trained to avoid crossover discussions and are blinded to the study aims and hypothesis. A single core multidisciplinary deprescribing team (pharmacist, nephrologist, dialysis nurse) will deliver the intervention across all participating clinics. This centralised model ensures consistency and minimises variability in practice. However, physicians outside the team are occasionally consulted (without revealing the trial hypothesis) if needed. Given the nature of the patients’ flow, we cannot guarantee the availability of all team members at each interaction with the patients. However, we standardise the intervention by providing training for all team members before the trial, periodic team meetings, shared standardised deprescribing forms and algorithms, and regular monitoring by the principal investigator.

### Randomisation, blinding, and allocation concealment

After obtaining informed consent, patients are randomised into one of the two study arms. Each recruited patient is assigned a unique code. Randomisation is stratified by dialysis dependency (dialysis vs. pre-dialysis) using randomly permuted balanced block varying block sizes to maintain allocation concealment.. The anticipated recruitment distribution is approximately 4:1 (dialysis: pre-dialysis), reflecting the underlying patient population at the study sites. The allocation sequence was generated by a separate computer and kept concealed from all research personnel. Once a patient is recruited and baseline data are collected, the assigned code is given by the responsible research assistant. Given the nature of the intervention, neither the healthcare provider delivering the intervention nor the patients can be blinded to the group allocation. To avoid contamination, the MDT-CKD physicians interact only with the participants in the intervention group.

### Deprescribing intervention and workflow

The proposed deprescribing workflow consists of the following stages:

1. Patient assessment: Upon recruitment, the patient’s information is collected through participant interviews and electronic medical records (via Cerner). These include sociodemographic, clinical, and medication information. Additionally, patients complete self-reported questionnaires to assess medication adherence, treatment burden, and quality of life.
2. Deprescribing plan development: For patients in the intervention group, the clinical pharmacist highlights the potentially problematic medications, drafts a deprescribing plan for each, consult the multidisciplinary team and the relevant specialists, and documents in the patient’s medical records. The potentially problematic medications are identified through the Medication Appropriateness Index (MAI) tool, and the deprescribing plans are drafted using deprescribing algorithms adapted from evidence-based deprescribing guidelines in the CKD setting (Zidan et al., 2025). Validation of these algorithms was conducted through input from an expert panel and piloted during the pilot RCT. The results of the validation and piloting are not yet published. The intervention targets all PIMs as we intend to provide a holistic approach. Prioritising the deprescribing plans is based on patient safety and medication properties. Only one medication is targeted per deprescribing cycle, unless deemed clinically appropriate. These are decided on case-by-case basis during the MDT-CKD meetings.
3. Implementation and monitoring of the deprescribing intervention: The team implements and monitors the approved deprescribing plan during the patient’s appointments at the centre or through follow-up phone calls, as applicable. The team periodically reviews the intervention’s progress, discussing results and addressing any issues to take corrective actions. Monitoring protocols are tailored per medication class as per the clinical guidelines.
4. Deprescribing plan finalisation: During subsequent appointments, the medication plan is reconciled and discussed with the patient. Patients are provided with a deprescribing card, containing updated medication information to ensure clear communication and adherence to the revised regimen.
5. Post-deprescribing follow-up: For each deprescribing plan, the MDT-CKD clinical pharmacist conducts three follow-up interviews or phone calls with the patients on days 2, 7, and 28 post-deprescribing. These follow-ups are designed to monitor late-onset withdrawal symptoms and to address any questions or concerns raised by the patient. Any changes to the patient’s medication regimen are documented and discussed with the multidisciplinary team to ensure appropriate management.
6. Documentation: All deprescribing processes and decisions related to each medication are documented in the participant’s medical records. For process evaluation, a checklist detailing each step of the deprescribing process is completed for every participant to ensure the fidelity of the intervention.

### Risk mitigation strategy

Although deprescribing is generally considered safe, both expected and unexpected risks will be managed in accordance with IRB SOP-09, which requires immediate reporting, review by the IRB, and corrective action if necessary. Furthermore, the deprescribing follow-up protocol was designed to actively monitor for immediate and late-onset adverse drug withdrawal events (ADWEs). Before plans implementation, anticipated risks are communicated to patients, and the monitoring plan is tailored accordingly. If an ADWE occurs, the deprescribing process may be slowed, modified, or discontinued depending on the specific medication, clinical plan, and patient response. In all cases, patients are provided with the direct contact numbers of the clinical team to report any concerns or sudden symptoms.

### Outcome measures

Outcomes will be assessed at different time points (baseline, 3, and 6 months) and compared between the two participant groups.

#### Primary outcome

***Potentially inappropriate medications (PIMs):*** This is represented as the number of events and non-events in each study group. It will also be reported as the percentage of participants with PIMs. PIMs are defined as drugs for which use should be avoided due to high risk of adverse reactions and/or insufficient evidence of benefit when safer and equally or more effective alternatives are available for this population (Fick et al., 2003). This will be determined through medical records using the MAI tool (Hanlon & Schmader, 2013). This implicit tool helps clinicians in making a comprehensive assessment of medication appropriateness. It contains 10 items to assess the appropriateness based on indication, efficacy, correct dosage, correct directions, practicality of the directions, interactions, duration of therapy, and costs. The medication is considered a PIM if the MAI score is ≥1. The MAI tool was deemed appropriate to identify PIMs among the target population as it provides a comprehensive overview of the prescribed medications. It has previously been used to identify PIMs among CKD patients (Wubshet H Tesfaye et al., 2019). In our assessment, the costs are not considered, given that the healthcare provider in Qatar (HMC) governs the decisions regarding the purchase of medication brands. Consequently, it was impractical for the research team to compare all medication alternatives (as the item of the cost mentioned in the MAI tool).

#### Secondary outcomes

- ***Pill burden:***Frequency and total number of daily medications, collected from medical records and confirmed during patient interviews. Pill burden was operationalised as a simple count of medications combined with their frequency of administration per day. This measure did not account for dosage forms or specific administration instructions.
- ***Deprescribing outcome:*** Total medications successfully discontinued, dose-reduced, substituted, or restarted after intervention (at 6-month follow-up), categorised by targeted pharmacological drug class.
- ***Health-related quality of life (HRQoL)****:* This will be measured using the self-administered Kidney Disease Quality of Life (KDQOL™) questionnaire (Hays et al., 1994).
- ***Treatment burden:*** This will be assessed using the Treatment Burden Questionnaire (TBQ) (Tran et al., 2014).
- ***Medication adherence****:* Assessed using the Adherence to Refills and Medications Scale (ARMS), a validated self-administered adherence measuring tool (Kripalani et al., 2009). Both English and Arabic versions of the tool are available for use in patients with chronic diseases (Alammari et al., 2021; Kripalani et al., 2009; Zidan et al., 2018).
- ***Hospitalizations and emergency department (ED) visits:*** Total number of hospitalisations and ED visits six months after the intervention. This will be extracted from medical records (CERNER).
- ***Cost–benefit and cost-savings:*** The return on investment in the proposed deprescribing intervention will be measured through cost–benefit analysis, considering cost-savings from drug use, hospitalisations, and ED visits compared to intervention costs.
- ***Unanticipated adverse events:*** Withdrawal symptoms (e.g. dyspepsia after stopping PPIs) or the need for drug re-initiation may be encountered in some cases. Each class of medications has different expected withdrawal symptoms. These or any other unanticipated events will be documented and reported to MRC-IRB as per IRB SOP-09.

#### Pilot stage outcomes

The following outcomes are measured during the ongoing pilot stage:

- Recruitment of ≥ 70% of eligible patients.
- Delivery of ≥ 75% of the intervention components.
- Retention rate of ≥75% at 6 months for outcome measures.
- No significant intervention-related safety concerns.

Furthermore, a detailed process evaluation is being conducted in accordance with MRC guidance [23] and will be published separately.

### Data collection instruments

- *Data collection form:* An Excel sheet was developed to collect demographic and clinical data and outcome measures. It contains participants’ characteristics, medical history, comorbidities, prescription information, laboratory data, and patient-reported outcomes.
- *Treatment Burden Questionnaire (TBQ):* The instrument’s responses are rated using a Likert-type scale ranging from 0 (not a problem) to 10 (a big problem). The TBQ global score is calculated by summation of each item score, with a maximum score of 150 points. The scores are further categorised as low burden (0–50), moderate burden (51–99), and high burden (100–150) (Tran et al., 2014).
- *Kidney Disease Quality of Life (KDQOL™) questionnaire:* The KDQOL-36^TM^ consists of five dimensions: Physical Component Summary (PCS), Mental Component Summary (MCS), Burdens of Kidney Disease (BKD), Symptoms and Problems of Kidney Disease (SPKD), and Effects of Kidney Disease (EKD). Numeric items are transformed into a 0–100 points scale, with total possible scores ranging from 0 to 3600 (higher scores indicate better quality of life) (Hays et al., 1994).
- *Adherence to Refills and Medications Scale (ARMS):* This is a 12-item medication adherence assessment tool with proven reliability and validity that was developed in English language and translated into several languages including Turkish,(Gökdoğan & Kes, 2017) Korean,(Kim et al., 2016) Chinese,(Chen et al., 2020) Polish,(Lomper et al., 2018) and Arabic.(Alammari et al., 2021; Zidan et al., 2018) The total scores range from 12 to 48, with higher scores indicating poor adherence (Kripalani et al., 2009).

### Data management and analysis plan

#### Sample size

Assuming normally distributed outcomes, comparing post-randomisation main outcomes between two groups, the number of subjects per group for a two-sided significance level (α) of 5% and power (1 – β) of 90% is 106. Considering the proportion of PIM (primary outcome) use among CKD patients is 40%, based on previously published studies (Tesfaye et al., 2017). Assuming a target decrease of 50% in the proportion of PIMs in the intervention group compared to the control group (Kua et al., 2019), a total of 212 participants (106 from each group) is needed. To account for a 10% dropout rate, a total of 250 participants will be needed, with 125 participants per group (Naing et al., 2022). While the initial calculation assumed a dropout rate of up to 50%, findings from the pilot study demonstrated that the actual dropout rate did not exceed 10%, supporting the feasibility of the adjusted sample size.

#### Statistical analysis plan

Data will be analyzed using SPSS® version 29. The statistical analysis will be based on the intention-to-treat principle (i.e. all available data of the recruited participants will be included in the analysis according to their allocated arms, including missing items due to withdrawal, death, or non-response). Data will be analyzed using both descriptive and inferential analyses. Continuous variables will be reported as median, mean, and standard deviation (SD), interquartile range (IQR), while categorical variables will be presented as frequencies and percentages. We will examine the descriptive statistics of costs, including mean, SD, minimum, and maximum, to detect any potential outliers prior to conducting a statistical comparison. Student *t*-test and Mann−Whitney *U*-test will be used for comparison of normally distributed and non-normally distributed variables, respectively.

#### Covariates and subgroups

Covariates for adjustment in all analyses will include the randomisation stratification variable, gender, comorbidities, and age. Baseline adjustment will be performed for the primary outcome and all relevant secondary outcomes.

#### Multiple testing

The analyses will perform multiple tests on the unadjusted and adjusted, and possibly sensitivity analyses of the primary outcome. No adjustment for multiple comparisons of secondary outcomes is planned since these are considered less critical than the primary outcome.

#### Detailed analyses approach

For all analyses, differences between treatment groups will be reported with 95% confidence intervals, and statistical significance will be assessed at the two-sided 0.05 level.

- Analysis of PIMs (primary outcome) between groups at 6 months will be performed using Poisson regression with adjustment of baseline values, age, comorbidities, and dialysis group. Secondary outcomes will be analyzed similarly, using either ordinary least squares or Poisson regression, as appropriate.
- Outcomes with repeated measures at 3 and 6 months, including the primary outcome, will be analyzed using mixed-effects models to account for correlated measurements within individuals over time. To assess differences between the two treatment groups, general linear models will include fixed effects for the treatment group, time point, and the interaction between the treatment group and the time point. Treatment effects will be reported separately at 6 months post-randomisation, regardless of the significance of the interaction term.
- For continuous outcomes, linear regression adjusted for baseline will be used without data transformations. Estimates of the treatment effect will be reported as mean differences in the change from baseline to 6 months between the intervention and control groups. For ordinal categorical outcomes, treatment effect estimates will be reported as proportional odds ratios for the intervention group compared with the control group using logistic regression. For nominal categorial (i.e. count) outcomes, treatment effects will be reported as rate ratios for the intervention group compared with the control group.
- Efforts will be made to minimise missing data. In case of encountering expected missing data, these will be documented and the reasons for missingness will be explored to enable the researchers to determine the proper imputation mechanism to estimate the missing values.

### Economic analysis plan

This will be performed from the perspective of the healthcare provider (i.e. HMC). The benefit-to-cost ratio (sum of cost savings and cost avoidance)/ (operational cost of the deprescribing plan), and the net benefit (sum of cost savings and cost avoidance) – (operational cost of the deprescribing plan) will be calculated.

- The total economic benefit of the deprescribing intervention will be calculated as the sum of the cost-savings resulting from medications use, and the cost avoidance associated with the intervention programme compared to usual care. Cost-savings based on the intervention will be the reduced cost of therapy associated with the reduction in utilisation (DDDs) of the targeted drugs, compared to the usual care. Cost avoidance will be the cost avoided by reducing ED visits and hospitalisations as unintended consequences of the polypharmacy use of medications.
- The cost of the programme will be represented by the operational cost of running the deprescribing intervention plan. The costs will include commonly considered non-protocol-driven resources that are strictly utilised under the different stages of the intervention plan workflow, including development and implementation. Also, the cost of the programme is any increase in the cost of medication use, hospitalisation, and ED visits, if any, compared to the usual care. Only direct medical costs will be considered in calculating the monetary value of the benefit associated with drug use, hospitalisation, and emergency admissions.

#### Sensitivity analysis

Sensitivity analysis will be used to enhance the robustness of the economic study conclusion and to enhance the generalizability of the study findings. The analysis tests will be used to assess modification on the base-case values of variables in relation to the costs and clinical probabilities. One-way and multivariate sensitivity analysis will be undertaken to analyze the effect of uncertain variables on the study conclusions. Both probabilistic analyses will be conducted via Monte Carlo simulation, using @Risk-7.6 (Palisade Corporation, NY).

### Trial monitoring and safety reporting

The trial will be monitored by the HMC-MRC and IRB according to their published Standard Operating Procedures (SOPs) provided to the investigators. These SOPs include, but are not limited to, processes of protecting human subjects related to reviewing and reporting unanticipated and serious adverse events.

## Discussion

The internal pilot study has confirmed the feasibility of the study processes with no identified safety concerns. The results from the pilot will be reported upon completion of the follow-up period. Recruitment is ongoing as planned, and the trial is expected to be completed by early 2026. The study will potentially provide critical evidence on both the clinical and economic benefits of deprescribing. It will also highlight the essential role of the clinical pharmacist within the multidisciplinary team providing care for patients with complex treatment regimens.

The trial is part of a larger work programme that includes developing deprescribing algorithms for CKD patients and a training programme for healthcare providers, which will be made publicly available. In addition, a comprehensive process evaluation is included in the trial; which would enable recommendations to be made about optimal implementation of deprescribing by multidisciplinary teams. Deprescribing is increasingly being recognised as an important intervention for various patient populations (Elbeddini et al., 2021; Thillainadesan et al., 2018; Triantafylidis et al., 2018). Therefore, the findings of this trial will be highly relevant to international and local healthcare settings by contributing to the growing body of evidence supporting deprescribing as a key strategy in optimising care for patients with chronic conditions such as CKD.

## Trial registration and protocol amendments

The trial was prospectively registered at ClinicalTrial.gov. NCT06324045, with outcome assessments at baseline and 6 months as specified in the original protocol. Shortly after recruitment commenced, an IRB-approved amendment (HMC-MRC: MRC-01-22-314/Amendment 02) added an interim 3-month assessment to characterise early effects and implementation. The 6-month endpoints and primary analyses were unchanged.
